# Supplementary material for: Differences between blacks and whites in well-being, beliefs, emotional states, behaviors and survival, 1978-2014
Source: PLoS One. 2020 Sep 14;15(9):e0238919. doi: 10.1371/journal.pone.0238919 (PMC7489510; doi:10.1371/journal.pone.0238919)

**Online Appendix**

Differences between blacks and whites in well-being, beliefs, emotional states, behaviors and survival, 1978-2014

**S1 Table.** Mean and standard deviation of well-being variables across different birth cohorts in the GSS-NDI

| **Birth Cohort** | **Age** | **Gender**  (male) | **Race**  (white) | **Income**  (above poverty line) | **Education**  (high school or above) | **Sex frequency**  (high sex frequency) | **Believing in God** (know God exists) | **Fair**  (depends or fair) | **Trust**  (depends or can trust) | **Happy**  (pretty or very happy) | **Health**  (good or excellent) |
| --- | --- | --- | --- | --- | --- | --- | --- | --- | --- | --- | --- |
|  | Mean  (SD) | N  (%) | N  (%) | N  (%) | N  (%) | N  (%) | N  (%) | N  (%) | N  (%) | N  (%) | N  (%) |
| **Prior to 1899** | 85.51 (3.42) | 55  (0.33) | 143  (0.85) | 1539  (0.86) | 60  (0.36) | - | 8  (0.80) | 89  (0.75) | 65  (0.57) | 143  (0.86) | 44  (0.45) |
| **1900** | 81.90 (4.20) | 97  (0.30) | 293  (0.90) | 3397  (0.84) | 126  (0.39) | 20  (0.71) | 19  (0.59) | 161  (0.69) | 93  (0.42) | 288  (0.89) | 113  (0.54) |
| **1905** | 78.65 (5.42) | 204 (0.33) | 541  (0.88) | 3148  (0.85) | 225  (0.37) | 73  (0.60) | 62  (0.68) | 271  (0.65) | 173  (0.43) | 532  (0.88) | 193  (0.50) |
| **1910** | 75.03 (6.35) | 347 (0.34) | 896  (0.88) | 2738  (0.86) | 497  (0.49) | 139  (0.61) | 126  (0.64) | 446  (0.64) | 304  (0.44) | 868  (0.88) | 410  (0.59) |
| **1915** | 72.59 (7.46) | 591 (0.39) | 1,341  (0.89) | 2134  (0.84) | 864  (0.58) | 242  (0.52) | 234  (0.64) | 617  (0.62) | 396  (0.40) | 1286 (0.89) | 561  (0.55) |
| **1920** | 68.34 (8.20) | 740 (0.40) | 1,667  (0.89) | 1685  (0.85) | 1,211  (0.65) | 344  (0.55) | 307  (0.65) | 779  (0.63) | 531  (0.44) | 1,587 (0.88) | 765  (0.59) |
| **1925** | 64.84 (8.70) | 810 (0.41) | 1,748  (0.88) | 489  (0.85) | 1,347  (0.68) | 428  (0.60) | 314  (0.60) | 786  (0.64) | 538  (0.42) | 1,650 (0.87) | 882  (0.65) |
| **1930** | 61.00 (8.96) | 854 (0.43) | 1,691  (0.86) | 1435  (0.85) | 1,464  (0.74) | 399  (0.53) | 363  (0.64) | 788  (0.64) | 541  (0.43) | 1,609 (0.88) | 940  (0.69) |
| **1935** | 55.75 (9.16) | 983 (0.45) | 1,883  (0.85) | 789  (0.84) | 1,732  (0.79) | 440  (0.54) | 400  (0.66) | 879  (0.63) | 613  (0.43) | 1,818 (0.89) | 1,060 (0.71) |
| **1940** | 51.20 (9.22) | 1,284 (0.45) | 2,487  (0.88) | 122  (0.81) | 2,372  (0.84) | 619  (0.56) | 516  (0.61) | 1124 (0.62) | 772  (0.41) | 2,318 (0.88) | 1,504 (0.78) |
| **1945** | 46.04 (9.21) | 1,642 (0.46) | 3,067  (0.86) | 258  (0.85) | 3,168  (0.89) | 738  (0.55) | 648  (0.63) | 1404 (0.61) | 947  (0.41) | 2,961 (0.89) | 1,924 (0.81) |
| **1950** | 41.06 (9.19) | 1,855 (0.45) | 3,489  (0.85) | 1512  (0.85) | 3,684  (0.90) | 918  (0.57) | 743  (0.61) | 1644 (0.62) | 1,115 (0.41) | 3,407 (0.88) | 2,297 (0.82) |
| **1955** | 36.61 (8.94) | 1,968 (0.44) | 3,751  (0.84) | 1189  (0.87) | 3,979  (0.89) | 1,025  (0.56) | 849  (0.64) | 1729 (0.62) | 1,168 (0.41) | 3,673 (0.88) | 2,510 (0.83) |
| **1960** | 33.18 (8.18) | 1,722 (0.45) | 3,192  (0.83) | 2960  (0.86) | 3,397  (0.89) | 987  (0.57) | 872  (0.66) | 1483 (0.63) | 1,017 (0.42) | 3,170 (0.89) | 2,297 (0.85) |
| **1965** | 30.65 (7.00) | 1,270 (0.45) | 2,302  (0.82) | 2104  (0.85) | 2,471  (0.88) | 834  (0.56) | 722  (0.64) | 1028 (0.61) | 739  (0.41) | 2,282 (0.90) | 1,682 (0.85) |
| **1970** | 28.68 (5.58) | 860 (0.44) | 1,636  (0.83) | 1505  (0.85) | 1,756  (0.89) | 654  (0.53) | 507  (0.63) | 699  (0.62) | 530  (0.42) | 1,538 (0.89) | 1,230 (0.85) |
| **1975** | 25.86 (4.25) | 619 (0.45) | 1,116  (0.82) | 1033  (0.86) | 1,190  (0.87) | 459  (0.56) | 314  (0.61) | 432  (0.61) | 357  (0.44) | 1,004 (0.88) | 816  (0.84) |
| **1980** | 23.84 (3.08) | 358 (0.43) | 669  (0.81) | 628  (0.86) | 702  (0.85) | 247  (0.54) | 275  (0.63) | 243  (0.62) | 190  (0.40) | 586  (0.86) | 454  (0.84) |
| **1985** | 21.11 (2.11) | 183 (0.44) | 323  (0.78) | 295  (0.82) | 345  (0.83) | 119  (0.50) | 205  (0.60) | 147  (0.59) | 108  (0.37) | 338  (0.88) | 205  (0.83) |

GSS-NDI: General Social Survey-National Death Index.

**S2 Figure.** Cohort effects on odds ratio of well-being variables for whites vs. blacks (reference: odds of well-being for blacks in the first cohort): (A) Self-rated health; (B) Happiness; (C) Trust; (D) Fair; (E) Belief in God; (F) Sex frequency; (G) Educational attainment with at least a high school degree; (H) Above poverty line.

**S3 Figure.** Cohort effects on hazard ratio of mortality for blacks vs. whites (reference was the hazard of mortality for whites in the first birth cohort): (A) Unadjusted mortality; and adjusted for (B) self-rated health; (C) happiness; (D) trust; (E) fair; (F) belief in God; (G) sex frequency; (H) High school degree; (I) Above poverty line.


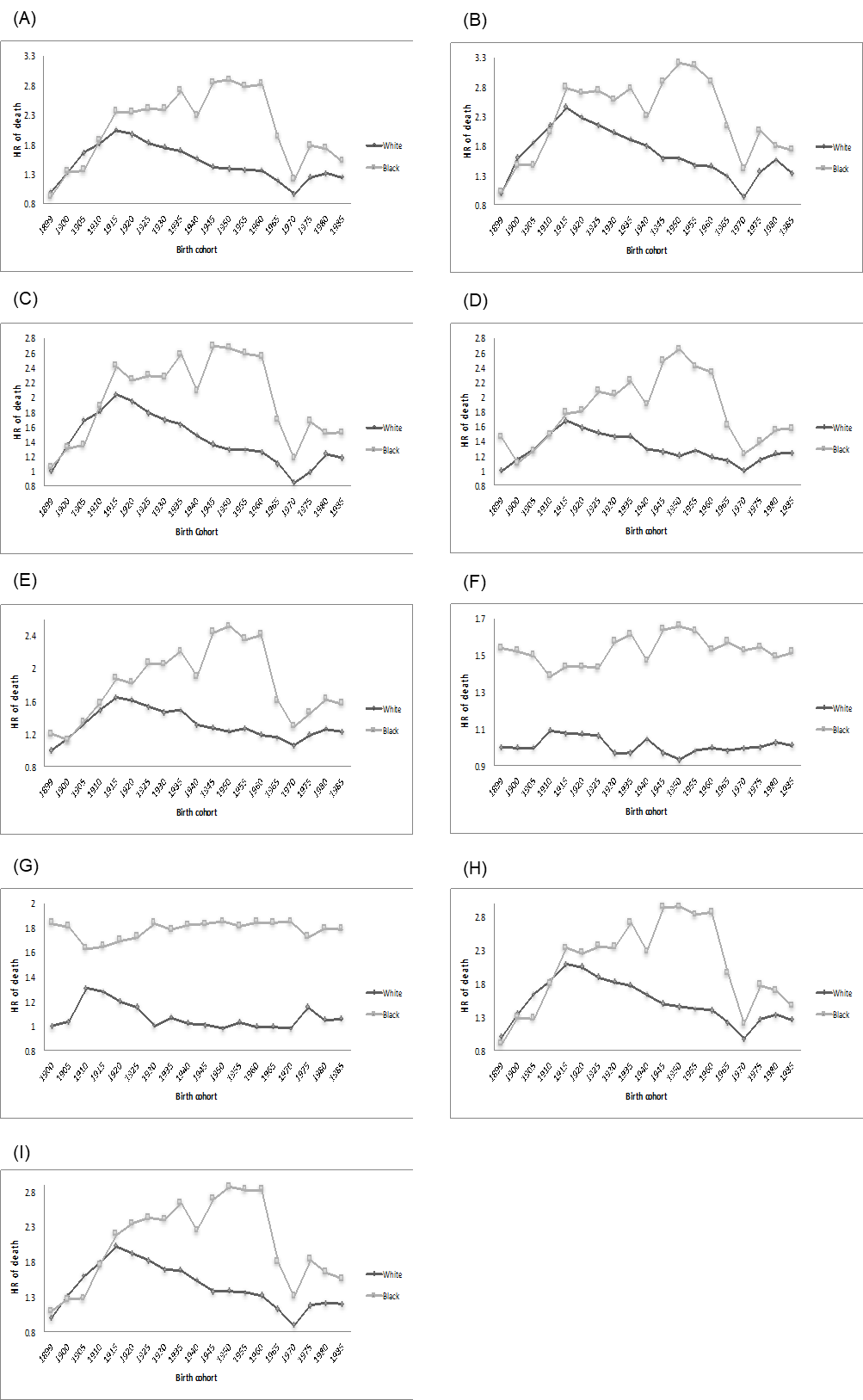

Supplement: S1 Appendix — (DOCX) [file pone.0238919.s001.docx]
